# Frog Virus 3 dissemination in the brain of tadpoles, but not in adult *Xenopus*, involves blood brain barrier dysfunction

**DOI:** 10.1038/srep22508

**Published:** 2016-03-02

**Authors:** Francisco De Jesús Andino, Letitia Jones, Sanjay B. Maggirwar, Jacques Robert

**Affiliations:** 1 Department of Microbiology and Immunology, University of Rochester Medical Center, Rochester, NY 14642, USA.

## Abstract

While increasing evidence points to a key role of monocytes in amphibian host defenses, monocytes are also thought to be important in the dissemination and persistent infection caused by ranavirus. However, little is known about the fate of infected macrophages or if ranavirus exploits immune privileged organs, such as the brain, in order to establish a reservoir. The amphibian *Xenopus laevis* and Frog Virus 3 (FV3) were established as an experimental platform for investigating *in vivo* whether ranavirus could disseminate to the brain. Our data show that the FV3 infection alters the BBB integrity, possibly mediated by an inflammatory response, which leads to viral dissemination into the central nervous system in *X. laevis* tadpole but not adult. Furthermore, our data suggest that the macrophages play a major role in viral dissemination by carrying the virus into the neural tissues.

The alarming increase in host range and amphibian mortality caused by ranaviruses (*Iridoviridae*) worldwide raises practical concerns about biodiversity and aquaculture, but also poses fundamental issues related to the evolution of host/pathogen interactions^1^,^2^,^3^,^4^,^5^. Ranaviruses, such as Frog virus 3 (FV3), are large double-stranded DNA viruses that infect fish, amphibians and reptiles^3^,^6^,^7^. Importantly, ranaviruses are capable of crossing species barriers among ectothermic vertebrates, suggesting that these pathogens possess potent immune evasion mechanisms^8^,^9^. Furthermore, although some species are highly susceptible to ranavirus, others are relatively resistant and can serve as asymptomatic carriers that disseminate infectious virus^10^,^11^, suggesting host specific factors which determine the course of infection.

Recent studies suggest a wider dissemination of FV3 through susceptible host species and, more specifically, within tadpoles compared to adult of the same species^12^. In addition, FV3 can persist quiescently in asymptomatic hosts^13^,^14^. The precise mechanisms of ranavirus dissemination within the host are still unclear. In this regard, we have proposed that the dissemination of FV3 may occur due to trafficking of infected macrophages into distal end-organs and tissues^15^. Interestingly, although ranavirus and FV3 in particular have been detected in various organs, including the kidneys, liver, intestine and spleen, the presence of infectious virus in the brain has not been reported. The brain is an immune privileged tissue, and so may provide a relatively safe environment for FV3 to establish a reservoir. Furthermore, FV3 infections are associated with inflammation that may cause tissue damage in tadpoles irrespective of viral load^16^, and such inflammation may compromise the blood brain barrier (BBB), which protects the brain from the rest of the body. In mammals, some viruses such as HIV can nevertheless bypass the BBB and infect brain tissues^17^,^18^,^19^. In addition, inflammation associated with viral infection has been shown to disrupt BBB integrity and allow leukocytes to penetrate the brain and cause damage^20^,^21^,^22^.

Although little is known about the BBB function in anuran amphibians, some studies suggest that it may be under-developed in tadpoles compared to adults. Since in *Xenopus* tadpoles, as compared to adults, FV3 is not efficiently contained in the kidney (the main site of infection in *Xenopus*), it was thought to ask if the brain of tadpoles is uniquely susceptible to FV3 infection.

## Results

### Dissemination of FV3 in the brain of *Xenopus laevis* tadpoles

**Figure 1:**
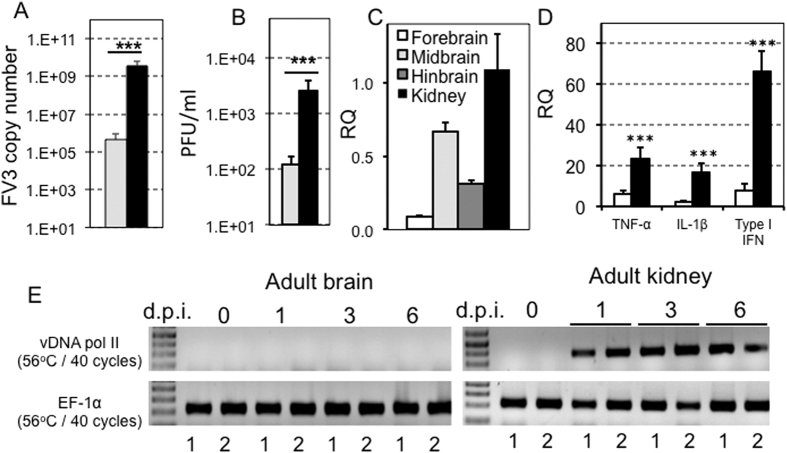
FV3 dissemination to the brain of tadpole but not adult *X. laevis*. Outbred pre-metamorphic tadpoles or adults were infected by i.p. injection of 1 × 10^4^ PFU or 1 × 10^6^ PFU of FV3, respectively. (**A**) FV3 genome copy number of tadpole brains (gray bars) and kidneys (black bars) at 6 d.p.i. (N = 6), determined by absolute qPCR using primers specific for FV3 vDNA Pol II. Results are means ± SE of the FV3 genome copy number per 50 ng of total DNA from 6 animals. ***P < 0.004 significant differences relative to tadpole brains by T-test. (**B**) Viral loads in tadpole brains (gray bars) and kidneys (black bars) at 6 d.p.i. (N = 6), determined by plaque assay. Results are representative of 3 replicates and displayed as means ± SE of the PFU/mL from 10 animals. ***P < 0.001 significant differences relative to tadpole’s brain by T-test. (**C**) Viral transcription in tadpole forebrain (white bars), midbrain (clear gray bar), hindbrain (dark gray bar) and kidneys (black bar) at 6 dpi animals, determined by qRT-PCR using primers specific for FV3 vDNA Pol II. P < 0.001 significant differences of each brain section relative to kidney using one-way ANOVA test and Tukey as post hoc test. There was no significant difference among the brain sections. (**D**) Change in the expression of the pro-inflammatory genes TNF-α and IL-1β as well as the antiviral gene type I IFN, in tadpole brains of 6 days post-FV3 (black bars) or sham-infected (white bars) animals, by qRT-PCR. Primers specific for *Xenopus* GAPDH (glyceraldehyde-3-phosphate dehydrogenase) were used as an endogenous control, and the expression of these genes were normalized to GAPDH. Results are means ± SE of gene expression from 6 animals. ***P < 0.001 significant differences relative to uninfected tadpole’s brain by T-test. (**E**) Detection of FV3 infection in two year-old *X. laevis* adult frog brains and kidneys (2 individuals per group) at 0, 1, 3 and 6 days post-infection. The presence of FV3 was detected in extracted DNA by PCR using primers specific for FV3 vDNA Pol II. EF-1α was use as a housekeeping gene control.

Although FV3 infects a wide variety of organs in animals following viral exposure, its dissemination to end organs, such as the brain, is unclear, particularly at the earliest stages of infection. In the present study we sought to determine whether FV3 disseminates into the brain of *Xenopus laevis* adults and tadpoles during the first week of infection. We chose to analyze viral spread in the brain at this time point, as it corresponds to the peak of FV3 infection and the corresponding immune response in both *X. laevis* tadpoles and adults^23^,^24^, and other viruses, such as HIV, are known to disseminate into the brain at the peak of viremia^25^,^26^. Accordingly, we harvested brain tissue from tadpoles and adults at 1 to 6 d.p.i. with FV3, and then assessed the presence of viral DNA by PCR. In several independent experiments FV3 DNA was consistently detected in tadpole brains at 6 d.p.i (Fig. 1A). Compared to tadpole kidneys the FV3 genome copy number in the brain was on average two orders of magnitude lower. However, the virus load was reliably found in all the brain samples tested and is likely to be the result of active viral replication since infectious particles were also detected in brain by plaque assays (Fig. 1B). In addition, FV3 was found to be transcriptionally active in tadpoles, as shown by the higher levels of vDNA Pol II gene expression in prominent regions of the brain (Fig. 1C). Additionally, the increased expression of several pro-inflammatory genes, including TNF-α, IL-1β, and type I IFN, in the brain of tadpoles (Fig. 1D) further implicates active viral replication. In contrast to tadpoles, however, FV3 was not detected in adult brain during the first week of infection (Fig. 1E), and there was no significant increase in pro-inflammatory gene expression in adult brain tissues (data not shown).

**Figure 2:**
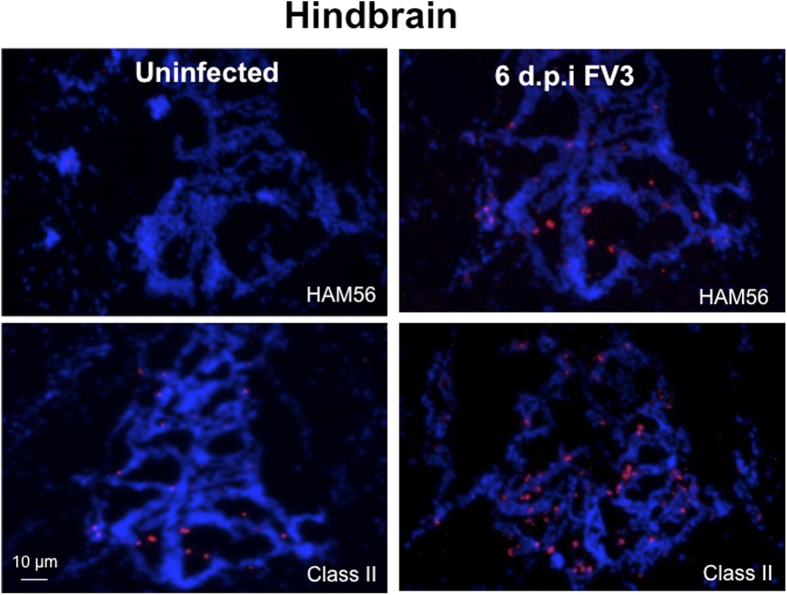
Accumulation of macrophage-like cells in FV3-infected tadpoles’ brain tissues. Cryosections (8 μm) of tadpole hindbrains 6 days post-FV3 infection were fixed with 4% cold paraformaldehyde then stained with *X. laevis* specific anti-class II AM20 or mouse anti-HAM56 mAbs followed by Dylight 594-conjugated F (ab’)_2_ donkey anti-mouse IgG (H + L) (Jackson Immuno Research, PA). Cellular nuclei were stained with the DNA intercalator Hoechst-33258. Sections were mounted in anti-fade medium (Molecular Probes, Oregon) and visualized with a fluorescence microscope using an Axiovert 200 inverted fluorescence microscope and Infinity 2 digital camera (objectives x5/x10/x20; Zeiss). Digital images were analyzed and processed by ImageJ software from NIH.

We have shown previously that the inflammatory response in the peritoneal cavity and kidney during FV3 infection is associated with the infiltration of myeloid lineage cells that are positive for the macrophage-specific marker HAM56 and express a higher level of MHC class II molecules^12^. Therefore, we asked whether a similar cellular response could be detected in the brains of infected tadpoles. Indeed, a high number of HAM56-positive cells were detected in the brain parenchyma of tadpoles at 6 d.p.i., whereas in uninfected tadpoles only a low number of HAM56-positive cells were observed (Fig. 2). Consistent with these results, an increased number of MHC class II-positive cells were also found in the brain of infected tadpoles as compared to uninfected control animals. Interestingly, and in agreement with the increased pattern of pro-inflammatory gene expression, we noted that the accumulation of macrophage-like cells was more prominent in the mid-brain region.

### Effect of FV3 infection on BBB function in *X. laevis* tadpoles

**Figure 3:**
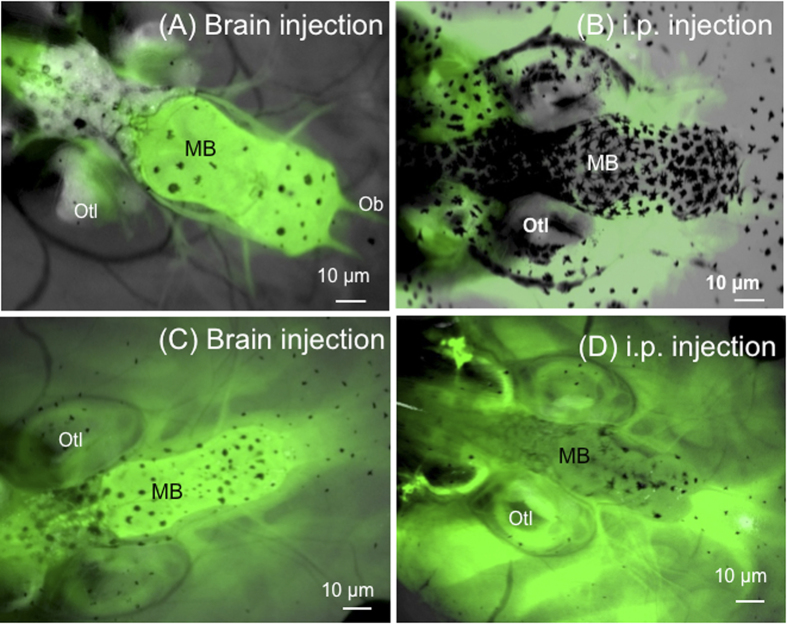
Functional measures of the BBB integrity in *X. laevis* tadpoles. Outbred pre-metamorphic uninfected tadpoles were injected in the brain with **(A)** 1 μg/mL (10 μL) NaF only or (**C**) NaF plus 1.36 M (5 μL) mannitol. Alternatively, tadpoles were injected in the intraperitoneal cavity with (**D**) NaF only or (**D**) NaF plus mannitol. Data shown are representative of 15–20 individuals. Diffusion of the tracer NaF was visualized by fluorescence microscopy as described in Fig. 2. MB, midbrain; Ob, olfactory bulb; Otl, otolift.

**Figure 4:**
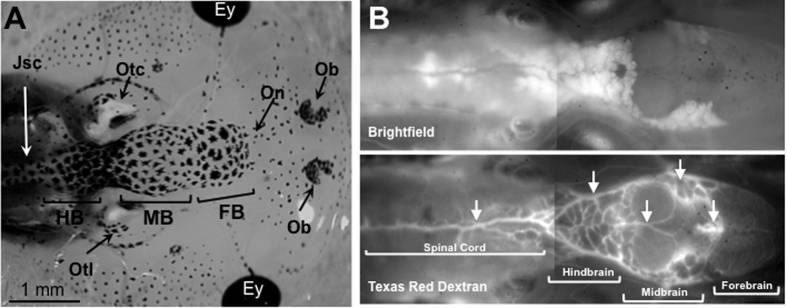
Visualization of tadpole’s brain and blood vasculature. (**A**) Dorsal **v**iew of a tadpole head at developmental stage 55 under a stereomicroscope at low magnification depicting the forebrain (FB), midbrain (MB), hindbrain (HB) and the junction with the spinal cord (JsC). Other anatomical structures visible are: Ey, eye; Ob, Olfactory bulb; On, olfactory neuron, Otc, Otocyst; Otl, Otolift. (**B**) Albinos outbred pre-metamorphic tadpoles were injected intracardially with Texas red dextran and 20 min later were anesthetized and observed under a fluorescent microscope with a low (5x) magnification objective. Arrows indicate major blood vessels.

To understand better how FV3 enters the brain, the functional integrity of the BBB in *X. laevis* tadpoles before and after infection with FV3 was investigated. Further, it was asked if the BBB is fully functional at this stage of development in tadpoles. A well-known assay with sodium fluorescein (NaF), which is a dye of small molecular weight (376 daltons) that can only cross the barrier paracellularly once it has been compromised^27^,^28^,^29^, was adapted for use in *X. laevis*. Pre-metamorphic tadpoles (3 weeks-old, stage 55) were injected with NaF either peripherally (i.p.) or directly into the brain, and its diffusion within a few hours across the barrier was examined by fluorescence microscopy. No leakage of NaF was detected in any combination (Fig. 3A,B; also see Fig. 4A for a view of the tadpole brain at low magnification), indicating that the BBB at this developmental stage is fully functional. To further confirm this notion, the BBB was disrupted by administration of mannitol (1.36 M) in tadpoles. This reagent has been previously shown to compromise the integrity of the BBB for a short time by altering osmotic pressure across the neurovascular unit in mammals^30^,^31^. As expected, within a few minutes of exposure to mannitol, extensive leakage of the NaF was detected in tadpoles (Fig. 3C,D). As shown in Fig. 4B, it appears that BBB leakage mainly occurs in the midbrain section where large blood vessels are located. These results suggest that the BBB is fully functional in tadpoles at stage 55.

**Figure 5:**
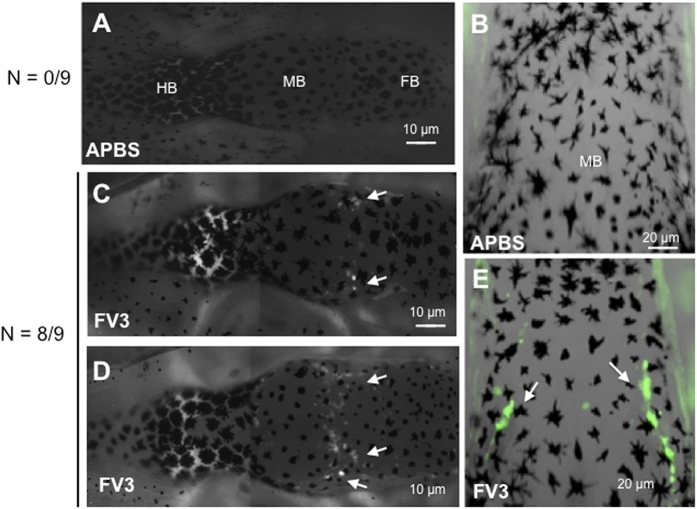
Dysfunction of the BBB during FV3 infection. Outbred pre-metamorphic tadpoles were sham-infected with APBS (**A**) whole brain and (**B**) midbrain view, or infected with 1 × 10^4^ PFU of FV3 by i.p. injection (**C,D**) whole brain and (**E**) midbrain view. At 6 d.p.i, tadpoles were i.p injected with 1 μg/mL (10 μL) NaF and the diffusion of the green fluorescent marker was visualized by fluorescence microscopy. Data shown are representative of 20 animals. FB, forebrain; MB, midbrain; HB, hindbrain.

Given the evidence that the BBB is fully functional in tadpoles at stage 55, we next assessed whether the BBB was compromised during FV3 infection. Tadpoles infected for 6 days were injected with NaF peripherally or in the brain. Compared to uninfected controls, infected tadpoles exhibited significant diffusion of NaF across the barrier indicative of BBB dysfunction (Fig. 5). Eight of nine infected tadpoles from 2 different experiments exhibited evidence of BBB leakage (Fig. 5C–E), whereas no uninfected tadpoles (n = 9) showed any sign of disruption (Fig. 5A,B). Interestingly, leakage in infected tadpoles appeared localized in discrete areas in the region between the forebrain and the midbrain (Fig. 5C–E, white arrows). To determine whether this phenomenon was associated with certain venules, tadpoles were injected with Texas Red Dextran (Fig. 4B).

### Infiltration of FV3-infected peritoneal leukocytes into the brain of *X. laevis* tadpoles

Previous studies in *X. laevis* have shown that FV3 particles are not typically present in the blood except during uncontrolled systemic infection. In contrast, peritoneal leukocytes (PLs) are readily infected by FV3 in tadpoles^12^. We have postulated that these cells, especially monocytes, can contribute to dissemination of ranavirus to different organs. This possibility was evaluated by conducting adoptive cell transfer experiments using the *X. laevis* MHC homozygous inbred strain J (j/j). Specifically, we asked whether adoptively transferred fluorescently labeled PLs could cross the BBB in tadpoles during FV3 infection.

**Figure 6:**
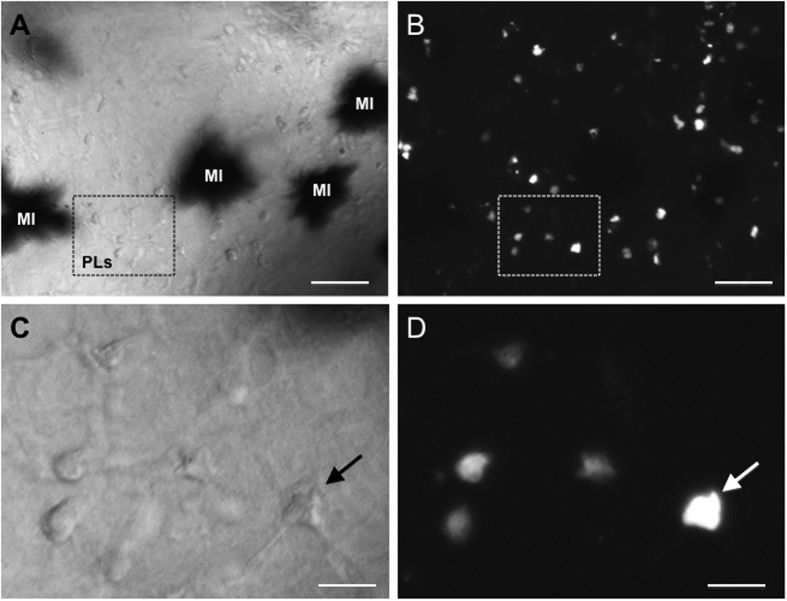
Peritoneal leukocyte transmigration across the BBB in tadpoles. Whole mount brain preparation from a tadpole at 6 d.p.i adoptively transferred by intracardiac injection of infected PLs labeled with PKH26. The midbrain area was examined under phase contrast (**A,C**) and fluorescence microscopy (**B,D**) at low magnification (10x objective) and high magnification (40x objective). The black spot in A are melanophores (MI). The arrow indicates the same cell. White bar =10 µm.

**Figure 7:**
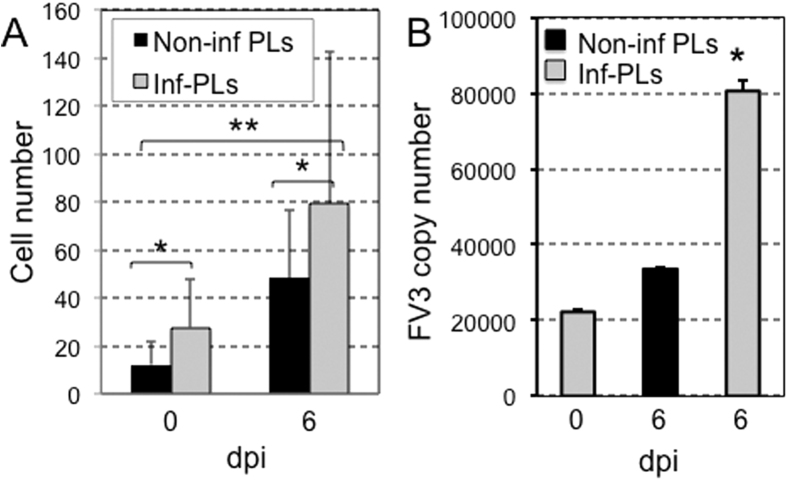
Infiltration of infected leukocytes and dissemination of FV3 in the tadpole’s brain. Mock-infected or 6 d.p.i FV3 pre-metamorphic inbred J tadpoles were adoptively transferred by intracardiac injection of 100,000 (10 μL volume) uninfected or *in vitro* FV3-infected (1 d.p.i) PLs previously labeled with 2 μM red fluorescent membrane PKH26. One day post-transfer, tadpole recipients were anesthetized and infiltration of leukocytes into the tadpole’s brain was visualized and quantified by fluorescence microscopy analysis of whole mount brain. **(A)** Results are representative of 3 replicates and displayed as means ± SE from 10 animals from uninfected tadpole recipients (0 dpi) injected with either uninfected (black bar) or infected (gray bar) PLs; or from infected tadpoles recipients at (6 dpi) injected either with uninfected (black bar) or infected (gray bar) PLs. *P < 0.05 and **P < 0.01 significant differences relative to uninfected tadpole’s brain using one-way ANOVA test and Tukey post hoc test. **(B)** FV3 genome copy number determined by absolute qPCR using primer specific for FV3 vDNAPol.

We collected PLs from adult J frogs that were stimulated with heat killed *E. coli*. Under these conditions, the PL population is primarily constituted by macrophages (75%) as previously shown^13^. These PLs were sham- or FV3-infected *in vitro* and cultured overnight. PL cultures were then labeled with PKH26 fluorescent membrane dye before adoptive transfer by intracardiac puncture into syngeneic tadpole recipients that were either uninfected or infected for 6 days with FV3. Infiltration of adoptively transferred cells in whole mount brain was monitored 24 h later by fluorescence microscopy on intact brain taking advantage of tadpole transparency (Fig. 6A,B). Observation of infiltrated cells at higher magnification confirmed their penetration into the brain parenchyma rather than in vascular circulation (Fig. 6C,D). In three independent experiments involving a total of 10 tadpole recipients for each group, large numbers of adoptively transferred PLs were found to infiltrate neural tissues in infected tadpole recipients as compared with their uninfected counterparts (Fig. 7A). Although it did not reach statistical significance because of individual variability, there was a trend toward increased infiltration of non-infected PLs in the brain of infected tadpoles consistent with the detection of large number of HAM56-positive cells found in infected tadpoles (Fig. 2).

Additional experiments were performed in which we infected, *in vitro*, the PLs derived from adult frogs and injected these cells into tadpoles that were either uninfected or infected for 6 d.p.i. with FV3. The migration of adoptively transferred PLs into the brain was measured as outlined above. As shown in Fig. 7A, a significantly higher number of infected PLs were found to transmigrate into the brain as compared to uninfected PLs. This pattern was more dramatic when the infected PLs were adoptively administered into the infected animals, suggesting that this may be a potential mechanism through which infectious virus gains entry into the brain. This notion was further confirmed in a parallel set of experiments in which FV3 copy number in brain specimens was measured by performing qPCR, and was also found to be increased dramatically (Fig. 7B).

Taken together, the data suggest that the PLs, possibly macrophages, harboring FV3 mediate viral entry into the tadpole brain by crossing the BBB and that the inflammation associated with FV3 infection may potentiate this phenomenon.

## Discussion

Increasing evidence points to a central role of monocytes in amphibian host defense as well as in the dissemination and persistence of ranavirus^15^,^24^,^32^. Here, we show that in *X. laevis* tadpoles FV3 infection alters the BBB integrity, possibly via triggering an inflammatory response, which leads to viral dissemination into the tadpole central nervous system. Furthermore, our data suggest that the macrophages play a major role in viral dissemination by carrying the virus into the neural tissues.

The presence of an effective BBB in adult frogs is well documented structurally^33^,^34^ and functionally^35^,^36^,^37^,^38^. However, it is not clear how early during the amphibian development the BBB matures and how efficiently it functions in anuran tadpoles. Some studies have been reported in zebrafish larvae, which suggest that the zebrafish BBB matures between 3 to 10 days post-fertilization (d.p.f.) and that this barrier shares both structural and functional similarities with that of mammals^39^. Our results indicate that the BBB is already mature enough to prevent leakage of NaF in immunocompetent *X. laevis* tadpoles at the pre-metamorphic developmental stage (stage 55). These results are consistent with recent findings in which the expression of claudin-5 and ZO-1 has been detected in brain postcapillary venules of zebrafish larva as early as 2 to 3 d.p.f.^40^,^41^. Analogous to our studies, these and other reports have employed fluorescent tracers to demonstrate that the size dependent exclusion occurs around 3 d.p.f. of zebrafish larvae^40^,^41^,^42^. However, size exclusion seems to occur only in certain brain microvessels of the zebrafish larvae at this age, while others are still “leaky”. In contrast to this, our results suggest that the *X. laevis* tadpoles possess tightly controlled BBB at stage 55, and the leakage of fluorescent tracer NaF following FV3 infection (6 d.p.i.) occurs mainly in the midbrain section where large blood vessels are located. In mammals, the BBB gradually matures during development, with permeability to small molecules decreasing with age^43^. Interestingly, as in mammals, we found that the osmotic diuretic mannitol rapidly disrupts the BBB in tadpoles, which could be easily visualized owing to the transparency of these animals.

Although the kidney is the main site of viral replication, FV3 dissemination into other organs is more prominent in *X. laevis* tadpoles than in adults where the infection is rapidly controlled and cleared^24^,^44^. Besides liver, spleen and lung, we show here that FV3 can also invade the central nervous system of tadpoles, which is not the case in adults. The detection of viral transcription and the increased expression of inflammatory genes in tadpole brain tissue suggest the presence of active viral infection. The spreading of FV3 infection into multiple tissues, including some more remote for an efficient immune response may in part explain why *X. laevis* tadpoles are unable to control FV3 and typically succumb from infection^24^,^44^. It is intriguing that FV3 does not infect *X. laevis* adult brain despite its persistence in a quiescent state in the kidney and in macrophages of asymptomatic animals^13^,^32^. It is possible that inflammation is better controlled (*i.e*. more localized and more short-lived) in adults than tadpoles, which in turn may minimize the negative impact on the BBB. Alternatively, the BBB in adult may be more efficient in preventing macrophage transmigration. Further investigation of developmental differences in BBB structure and function will be needed to resolve this issue.

Increasing evidence indicates that higher susceptibility of tadpoles to ranavirus infection is not limited to *X. laevis*^45^. However, the increased susceptibility of tadpoles is not simply due to a lack of immune response but likely involves strategies employed by ranavirus to evade and counteract host immune responses. For example, although *X. laevis* tadpoles mount a timely and robust type III IFN-l response, it is efficiently overcome by FV3 possibly via a rapid down regulation of the IFN-l receptors^46^. Our present study further suggests that FV3 may take advantage of the mobility of macrophages and the inflammation elicited during infection to disseminate throughout the organism, including to the central nervous system. Our adoptive transfer experiments indicate that the disrupted BBB of infected tadpoles is more permissive to the infiltration of PLs. Also interesting is the fact that the infected PLs are able to infiltrate the brain more efficiently than uninfected PLs, which suggests a strong possibility that the inflammatory factor released by these cells may promote their passage through the BBB. This may be analogous to the transmigration of PLs observed in models of other viral infections, including HIV-1^29^,^47^,^48^,^49^.

Disruption of the BBB and dissemination of viral infection is an active research field in mammals for HIV^22^,^49^ and other virus^50^,^51^,^52^. As such, the *X. laevis* tadpole may provide a useful new model system to investigate the modalities of this phenomenon. The ease by which the BBB integrity can be assessed by simple microscopic observation is an attractive feature of *X. laevis* tadpole. Its transparency, relative small size and resilience to room temperature, as well as the accessibility of the central nervous system for experimentation, and the relative low cost for testing large numbers of individuals, make *X. laevis* tadpole an ideal model for intravital investigation of the BBB.

## Materials and Methods

### Animals

Outbred (OB) young adults (2 years old) and pre-metamorphic tadpoles (stage 54–56/3 weeks-old), as well as MHC homozygous inbred strain J (j/j) pre-metamorphic J tadpoles and young adult frogs were obtained from our *X. laevis* research resource for immunobiology at the University of Rochester (https://www.urmc.rochester.edu/microbiology-immunology/xenopus-laevis.aspx).

Experiments involving tadpoles and frogs were carried out according to the Animal Welfare Act from the United States Department of Agriculture (USDA), the Public Health Service Policy (A-3292-01) and the Public Health Act of New York State. Any discomfort was minimized at all time. Animal care and all the protocols have been reviewed and approved by the University of Rochester Committee on Animal Resources (Approval number 100577/2003-151).

### Cell lines, FV3 Stocks and Animal Infections

Baby hamster kidney-21 cells (BHK-21; ATCC no. CCL-10) were maintained in Dulbecco’s modified Eagle’s medium (DMEM; Invitrogen) supplemented with 10% fetal bovine serum (FBS; Invitrogen), penicillin (100 U/mL) and streptomycin (100 μg/mL) with 5% CO_2_ at 37 °C. FV3 was grown by a single passage on BHK-21 cells, purified by ultracentrifugation on a 30% sucrose gradient and quantified by plaque assay on BHK-21 monolayers under an overlay of 1% methylcellulose^13^.

Tadpoles were infected by intraperitoneal (i.p.) injection with 1 × 10^4^ PFU of FV3 in 10 μL volume using a glass Pasteur pipette whose small end had been pulled in a flame. Adult frogs were infected by i.p. injection with 1 × 10^6^ PFU of FV3 in 100 μL volume using a 1 ml sterile syringe with a 22 gauge, 1½ inch needle. Controls (0 days post infection, d.p.i.) were mock-infected with the same amount of amphibian phosphate-buffered saline (APBS). At different time points, tadpoles (0 and 6 d.p.i.) or adult frogs (0, 1, 3 and 6 d.p.i.) were euthanized by immersion in 1% tricaine methane sulfonate (TMS-222) buffered with bicarbonate.

### Sodium Fluorescein (NaF) assays

Uninfected tadpoles were injected i.p. with 1 μg/mL (in a total volume of 10 μL) green fluorescent tracer NaF in absence or presence of mannitol (1.36 M in a total volume of 5 μL, also injected i.p.). In this case mannitol was used as a positive control that is known to disrupt BBB osmotically^30^,^31^. In parallel, some uninfected tadpoles received microinjection of NaF and mannitol directly in the brain. Briefly this was done by injection either in the brain by microinjection (Pico-Injector Microinjection System, Harvard Apparatus) or i.p. After 10–30 minutes the diffusion of the NaF with or without mannitol was visualized by fluorescence microscopy as a marker of BBB dysfunction.

Additional experiments were performed in which NaF was administered (i.p.) in sham-infected or infected (6 d.p.i.) pre-metamorphic tadpoles. After the overnight incubation, the cerebral diffusion of NaF was visualized by a fluorescence microscopy.

All specimens were examined using an Axiovert 200 inverted fluorescence microscope and Infinity 2 digital camera (objectives x5/x10; Zeiss). Digital images were analyzed and processed by ImageJ software from NIH (URL: http://imagej.nih.gov/ij/).

### Plaque Assays

Brain and kidney tissues were homogenized in hypotonic buffer (Tris-HCL 50 mM; pH 7.5) by 3 freeze/thaw cycles and serially diluted in DMEM supplemented with 2.5% FBS (Grayfer, L. *et al*., 2014). Five hundred microliters of each dilution was plated in duplicate on BHK-21 confluent monolayer in 6 well plates at room temperature for 1 hour. Supernatant were removed by aspiration, and 3 mL of overlay medium (DMEM supplemented with 2.5% FBS and 1% Methyl cellulose; Sigma) was added. Cells were incubated for 6 days at 37 °C in 5% CO_2_. Overlay medium was aspirated, and the cells were stained for 10 minutes with 1% crystal violet in 20% ethanol.

### Gene expression analysis

Total RNA was isolated from tadpole and adult frog tissues using Trizol reagent (Invitrogen) following the manufacturer’s protocol. Conventional methods were also used to isolate DNA from tissues derived from tadpoles and adult frogs. RNA (10 μg) was digested with DNAse (Ambion, Life Technologies) and used to synthetized cDNA with the iScript cDNA synthesis kit (Bio-Rad, Hercules, CA) that contained other reagents including oligo dT, dNTPs, random hexamer primers and reverse transcriptase (Invitrogen). cDNA (1 μL) template was used in all reverse transcriptase (RT)-polymerase chain reactions (PCRs), whereas 100 ng DNA was used for PCR. Minus RT (-RT) controls, for DNA contamination, were included for every reaction and generated for each sample analogous to cDNA that was devoid of active RT. RT-PCR and PCR products were separated on 1.0% agarose gels and stained with ethidium bromide. Sizes of nucleic acid products were determined using standardized markers of 1 kb plus from Invitrogen (Carlsbad, CA). All primers are listed in Table 1.

For quantitative PCR (qPCR), 2.5 μL of (1:2) diluted cDNA or genomic DNA (150 ng) was amplified in a mixture of 10 μL containing 200 nM of each primer and 1× SYBR green FastMix containing 1× ROX passive reference dye. Relative qPCR expression (RQ) was examined using the delta delta CT threshold cycle (CT) method, with the level of expression compared relative to the glyceraldehyde-3-phospahte dehydrogenase (GAPDH) endogenous control and normalized to the lowest observed level of expression (Grayfer *et al*. 2014). To measure the viral load and viral DNA Polymerase II (vDNA Pol II) gene expression, absolute qPCR analysis was performed on DNA using a serially diluted standard curve. Briefly, FV3 vDNA Pol II fragment was cloned, amplified, quantified, and diluted between 10^10^ to 10^1^ plasmid copies of vDNA Pol II. These dilutions were used to create a standard curve to further determine the vDNA Pol II transcript copy numbers relative to those on the standard curve (Grayfer *et al*. 2014). All qPCR analyses were performed using ABI 7300 real-time PCR system and PerfeCTa® SYBR Green DastMix, ROX (Quanta). Relative expression was estimated by using ABI sequence detection system software (SDS). All primers were validated prior to use. Each sample was run in two replicates. Melting curve analysis was carried out after each PCR run to ensure the specificity of the reaction.

### Bacterial stimulation

*E. coli* (XL1-blue, Strategene, La Jolla, Ca.) cultured overnight at 37 °C, were boiled for 1 hour, centrifuged and resuspended in 0.1 volume (approximately 10^8^ bacteria/ml) of *Xenopus* cell culture medium^53^. Frogs were injected i.p. with 300 μL of heat-killed bacteria (HK *E. coli*) mixture (3 × 10^7^ bacteria; corresponding to 3 mg of protein). After 3 days post stimulation, peritoneal leukocytes (PLs) were removed by peritoneal lavage with sterile APBS, washed, quantified and cultured in *Xenopus* medium amphibian serum free plus 10% FBS (ASF + 10% FBS).

### Infiltration of immune cells labeled with PKH26 red dye

PLs were collected from inbred J young adult frogs that were treated with bacterial stimuli and purified cells were cultured for 1day in Iscove-derived amphibian culture medium supplemented with 10% FBS^54^. These cells were either sham- infected *in vitro* or infected with FV3 (1 multiplicity of infection, MOI). After 1 d.p.i, the cells were washed with sterile APBS, quantified and labeled with the red fluorescent membrane dye PKH26 (Sigma-Aldrich) (Invitrogen) according to the manufacturer’s protocol. Briefly, the cell suspension was mixed with an equal volume of the labeling solution containing 2 μM PKH26 in the dilution buffer and incubated for 5 to 20 minutes at 27 °C, respectively. The reaction was terminated by adding 5 mL *Xenopus* culture medium, and cells were washed extensively with sterile APBS. One hundred thousand PLs uninfected or infected for 1day with FV3 cells were adoptively transferred into uninfected or FV3-infected (6 d.p.i) pre-metamorphic inbred J tadpoles by i.p. injection. One day post-transfer, tadpoles were anesthetized with 0.1% TMS and the intact brain regions were analyzed by fluorescence microscopy. In another group of tadpoles, blood vessels were stained by intercardiac microinjection with 25 mg/mL (10 μL) Texas Red Dextran (Sigma-Aldrich). After 5–10 minutes incubation, tadpoles were anesthetized and blood vessels/labeled PL cells were tracked by fluorescence microscopy.

All experimental specimens were examined by using an Axiovert 200 inverted fluorescence microscope and Infinity 2 digital camera (objectives x5/x10/x20; Zeiss). Digital images were analyzed and processed by ImageJ software from NIH (URL: http://imagej.nih.gov/ij/).

### Immunohistochemial Analyses

Sham-infected or infected (6 d.p.i.) outbred pre-metamorphic tadpoles were euthanized in 1% TMS, incubated in 8% sucrose overnight and embedded into Optimal Cutting Temperature compound (OCT) for 8 μm cryosections. Sections were sequentially rehydrated in APBS for 10 minutes, fixed with 4% cold paraformaldehyde (4 °C) for 15 minutes, permeabilized with 100% cold methanol (−20 °C) for 5 minutes and washed extensively with APBS (25 °C) for 5 minutes. After blocking with 1% BSA +0.2% Tween 20 in APBS for 2 hour at room temperature, the sections were incubated overnight with the mouse monoclonal antibody HAM56 (Abcam ab45018-500) or anti-MHC class II monoclonal antibody AM20 hybridoma supernatant (Produced and validated in our X. laevis research resource for immunobiology). After washing, cells were incubated with Dylight 594-conjugated F (ab’)_2_ donkey anti-mouse IgG (H + L) (Jackson Immuno Research, PA). Cellular nuclei were then stained with the DNA intercalator Hoechst-33258. Sections were mounted in anti-fade medium (Molecular Probes, Oregon) and visualized with a fluorescence microscope using an Axiovert 200 inverted fluorescence microscope and Infinity 2 digital camera (objectives x5/x10/x20; Zeiss). Digital images were analyzed and processed by ImageJ software from NIH (URL: http://imagej.nih.gov/ij/).

### Statistical analysis

Quantitative data were analyzed using either a T-test or a One-Way Analysis of Variance (ANOVA) for independent or correlated samples and performed using an online database available through Vassar Stat a website for statistical computation (URL: http://vassarstats.net/anova1u.html).

## Additional Information

**How to cite this article**: Francisco, D. J. A. *et al*. Frog Virus 3 dissemination in the brain of tadpoles, but not in adult *Xenopus*, involves blood brain barrier dysfunction. *Sci. Rep*. **6**, 22508; doi: 10.1038/srep22508 (2016).

## Figures and Tables

**Table 1:** List of primer sequences.

PRIMER	SEQUENCE (5′-3′)
	**CONVENTIONAL PRIMERS**
EF-1α	F: CCTGAATCACCCAGGCCAGATTGGTGR: GAGGGTAGTGTGAGAAGCTCTCCACG
FV3 DNA Poly II	ACGAGCCCGACGAAGACTACATAGTGGTGGTCCTCAGCATCCTTTG
	**Q-PCR PRIMERS**
FV3 DNA Poly II	F: ACGAGCCCGACGAAGACTACAR: TGGTGGTCCTCAGCATCC T
GAPDH	F: GACATCAAGGCCGCCATTAAGACTR: AGATGGAGGAGTGAGTGTCACCAT
IL-1β	F: CATTCCCATGGAGGGCTACAR: TGACTGCCACTGAGCAGCAT
Type I IFN	F: GCTGCTCCTGCTCAGTCTCAR: GAAAGCCTTCAGGATCTGTGTGT
TNF-α	F: TGTCAGGCAGGAAAGAAGCAR: - CAGCAGAGCAAAGAGGATGGT

F: Forward; R: Reverse.

## References

[b1] Chen G. & Robert J. Antiviral immunity in amphibians. Viruses 3, 2065–2086, doi: 10.3390/v3112065 (2012). 22163335 PMC3230842

[b2] Robert J. & Gregory Chinchar V. “Ranaviruses: an emerging threat to ectothermic vertebrates” report of the First International Symposium on Ranaviruses, Minneapolis MN July 8, 2011. Dev Comp Immunol 36, 259–261, doi: 10.1016/j.dci.2011.08.008 (2012). 21906623

[b3] Gray M. J., Miller D. L. & Hoverman J. T. Ecology and pathology of amphibian ranaviruses. Dis Aquat Organ 87, 243–266 (2009). 20099417 10.3354/dao02138

[b4] Collins J. P. Amphibian decline and extinction: what we know and what we need to learn. Dis Aquat Organ 92, 93–99, doi: 10.3354/dao02307 (2010). 21268970

[b5] Daszak P. . Emerging infectious diseases and amphibian population declines. Emerg Infect Dis 5, 735–748 (1999). 10603206 10.3201/eid0506.990601 PMC2640803

[b6] Chinchar V. G., Yu K. H. & Jancovich J. K. The molecular biology of frog virus 3 and other iridoviruses infecting cold-blooded vertebrates. Viruses 3, 1959–1985, doi: 10.3390/v3101959 (2011). 22069524 PMC3205390

[b7] Green D. E., Converse K. A. & Schrader A. K. Epizootiology of sixty-four amphibian morbidity and mortality events in the USA, 1996–2001. Ann N Y Acad Sci 969, 323–339 (2002). 12381613 10.1111/j.1749-6632.2002.tb04400.x

[b8] Johnson A. J. . Ranavirus infection of free-ranging and captive box turtles and tortoises in the United States. J Wildl Dis 44, 851–863, doi: 44/4/851 (2008). 18957641 10.7589/0090-3558-44.4.851

[b9] Mao J., Green D. E., Fellers G. & Chinchar V. G. Molecular characterization of iridoviruses isolated from sympatric amphibians and fish. Virus Res 63, 45–52 (1999). 10509715 10.1016/s0168-1702(99)00057-x

[b10] Hoverman J. T., Gray M. J., Haislip N. A. & Miller D. L. Phylogeny, life history, and ecology contribute to differences in amphibian susceptibility to ranaviruses. Ecohealth 8, 301–319, doi: 10.1007/s10393-011-0717-7 (2011). 22071720

[b11] Teacher A. G., Garner T. W. & Nichols R. A. Evidence for directional selection at a novel major histocompatibility class I marker in wild common frogs (Rana temporaria) exposed to a viral pathogen (Ranavirus). PloS one 4, e4616, doi: 10.1371/journal.pone.0004616 (2009). 19240796 PMC2643007

[b12] Robert J., George E., De Jesus Andino F. & Chen G. Waterborne infectivity of the Ranavirus frog virus 3 in Xenopus laevis. Virology 417, 410–417, doi: 10.1016/j.virol.2011.06.026 (2011). 21783222 PMC3163690

[b13] Morales H. D. . Innate immune responses and permissiveness to ranavirus infection of peritoneal leukocytes in the frog Xenopus laevis. J Virol 84, 4912–4922, doi: 10.1128/JVI.02486-09 (2010). 20200236 PMC2863837

[b14] Robert J., Abramowitz L., Gantress J. & Morales H. D. Xenopus laevis: a possible vector of Ranavirus infection? J Wildl Dis. 43, 645–652 (2007). 17984259 10.7589/0090-3558-43.4.645

[b15] Grayfer L., Andino Fde J., Chen G., Chinchar G. V. & Robert J. Immune evasion strategies of ranaviruses and innate immune responses to these emerging pathogens. Viruses 4, 1075–1092, doi: 10.3390/v4071075 (2012). 22852041 PMC3407895

[b16] Grayfer L., De Jesus Andino F. & Robert J. The amphibian (Xenopus laevis) type I interferon response to frog virus 3: new insight into ranavirus pathogenicity. J Virol 88, 5766–5777, doi: 10.1128/jvi.00223-14 (2014). 24623410 PMC4019129

[b17] Nakaoke R., Ryerse J. S., Niwa M. & Banks W. A. Human immunodeficiency virus type 1 transport across the *in vitro* mouse brain endothelial cell monolayer. Exp Neurol 193, 101–109, doi: 10.1016/j.expneurol.2004.11.020 (2005). 15817268

[b18] Dohgu S. & Banks W. A. Brain pericytes increase the lipopolysaccharide-enhanced transcytosis of HIV-1 free virus across the *in vitro* blood-brain barrier: evidence for cytokine-mediated pericyte-endothelial cell crosstalk. Fluids Barriers CNS 10, 23, doi: 10.1186/2045-8118-10-23 (2013). 23816186 PMC3710206

[b19] Dohgu S., Ryerse J. S., Robinson S. M. & Banks W. A. Human immunodeficiency virus-1 uses the mannose-6-phosphate receptor to cross the blood-brain barrier. PloS one 7, e39565, doi: 10.1371/journal.pone.0039565 (2012). 22761827 PMC3382565

[b20] Ramirez S. H. . Dyad of CD40/CD40 ligand fosters neuroinflammation at the blood-brain barrier and is regulated via JNK signaling: implications for HIV-1 encephalitis. J Neurosci. 30, 9454–9464, doi: 10.1523/jneurosci.5796-09.2010 (2010). 20631174 PMC2908988

[b21] Yang B., Akhter S., Chaudhuri A. & Kanmogne G. D. HIV-1 gp120 induces cytokine expression, leukocyte adhesion, and transmigration across the blood-brain barrier: modulatory effects of STAT1 signaling. Microvasc Res 77, 212–219, doi: 10.1016/j.mvr.2008.11.003 (2009). 19103208 PMC3715090

[b22] Williams D. W., Anastos K., Morgello S. & Berman J. W. JAM-A and ALCAM are therapeutic targets to inhibit diapedesis across the BBB of CD14^+^CD16^+^monocytes in HIV-infected individuals. J Leukoc Biol. 97, 401–412, doi: 10.1189/jlb.5A0714-347R (2015). 25420915 PMC4304417

[b23] Chen G. & Robert J. Antiviral immunity in amphibians. Viruses 3, 2065–2086, doi: 10.3390/v3112065 (2011). 22163335 PMC3230842

[b24] De Jesus Andino F., Chen G., Li Z., Grayfer L. & Robert J. Susceptibility of Xenopus laevis tadpoles to infection by the ranavirus Frog-Virus 3 correlates with a reduced and delayed innate immune response in comparison with adult frogs. Virology 432, 435–443, doi: 10.1016/j.virol.2012.07.001 (2012). 22819836 PMC3574294

[b25] Hurtrel B. . Early SIV encephalopathy. J Med Primatol. 20, 159–166 (1991). 1942006

[b26] Peterson J. . Cerebrospinal fluid (CSF) neuronal biomarkers across the spectrum of HIV infection: hierarchy of injury and detection. PloS one 9, e116081, doi: 10.1371/journal.pone.0116081 (2014). 25541953 PMC4277428

[b27] Seelbach M. . Polychlorinated biphenyls disrupt blood-brain barrier integrity and promote brain metastasis formation. Environ Health Perspect 118, 479–484, doi: 10.1289/ehp.0901334 (2010). 20064788 PMC2854723

[b28] Ramirez S. H. . Activation of cannabinoid receptor 2 attenuates leukocyte-endothelial cell interactions and blood-brain barrier dysfunction under inflammatory conditions. J Neurosci 32, 4004–4016, doi: 10.1523/jneurosci.4628-11.2012 (2012). 22442067 PMC3325902

[b29] Davidson D. C. . Excess soluble CD40L contributes to blood brain barrier permeability *in vivo*: implications for HIV-associated neurocognitive disorders. PloS one 7, e51793, doi: 10.1371/journal.pone.0051793 (2012). 23251626 PMC3520914

[b30] Pappius H. M., Savaki H. E., Fieschi C., Rapoport S. I. & Sokoloff L. Osmotic opening of the blood-brain barrier and local cerebral glucose utilization. Ann Neurol 5, 211–219 (1979). 443753 10.1002/ana.410050302

[b31] Birngruber T. . Cerebral open flow microperfusion: a new *in vivo* technique for continuous measurement of substance transport across the intact blood-brain barrier. Clin Exp Pharmacol Physioly 40, 864–871, doi: 10.1111/1440-1681.12174 (2013). 24256164

[b32] Robert J., Grayfer L., Edholm E. S., Ward B. & De Jesus Andino F. Inflammation-induced reactivation of the ranavirus Frog Virus 3 in asymptomatic Xenopus laevis. PloS one 9, e112904, doi: 10.1371/journal.pone.0112904 (2014). 25390636 PMC4229299

[b33] Rascher G. & Wolburg H. The tight junctions of the leptomeningeal blood-cerebrospinal fluid barrier during development. Journal fur Hirnforschung 38, 525–540 (1997). 9476217

[b34] Lazzari M., Bettini S., Ciani F. & Franceschini V. Glucose transporter distribution in the vessels of the central nervous system of the axolotl Ambystoma mexicanum (Urodela: Ambystomatidae). Anat Rec (Hoboken) 291, 1293–1300 (2008). 18727107 10.1002/ar.20741

[b35] Li L., Wang W., Lv Q., Ben Y. & Li X. Bioavailability and tissue distribution of Dechloranes in wild frogs (Rana limnocharis) from an e-waste recycling area in Southeast China. J Environ Sci (China) 26, 636–642, doi: 10.1016/s1001-0742(13)60447-7 (2014). 25079277

[b36] Fraser P. A. & Dallas A. D. Permeability of disrupted cerebral microvessels in the frog. J Physiol 461, 619–632 (1993). 8350276 10.1113/jphysiol.1993.sp019532 PMC1175276

[b37] Nagy Z., Pettigrew K. D., Meiselman S. & Brightman M. W. Cerebral vessels cryofixed after hyperosmosis or cold injury in normothermic and hypothermic frogs. Brain Res 440, 315–327 (1988). 3258781 10.1016/0006-8993(88)91001-3

[b38] Jones H. C. & Taylor C. M. Absorption of the cerebrospinal fluid and intracranial compliance in an amphibian, Rana pipiens. J Physiol 353, 405–417 (1984). 6207286 10.1113/jphysiol.1984.sp015343 PMC1193314

[b39] Fleming A., Diekmann H. & Goldsmith P. Functional characterisation of the maturation of the blood-brain barrier in larval zebrafish. PloS one 8, e77548, doi: 10.1371/journal.pone.0077548 (2013). 24147021 PMC3797749

[b40] Jeong J. Y. . Functional and developmental analysis of the blood-brain barrier in zebrafish. Brain Res Bull 75, 619–628, doi: 10.1016/j.brainresbull.2007.10.043 (2008). 18355638

[b41] Xie J., Farage E., Sugimoto M. & Anand-Apte B. A novel transgenic zebrafish model for blood-brain and blood-retinal barrier development. BMC Dev Biol 10, 76, doi: 10.1186/1471-213x-10-76 (2010). 20653957 PMC2914679

[b42] Tam S. J. . Death receptors DR6 and TROY regulate brain vascular development. Dev Cell 22, 403–417, doi: 10.1016/j.devcel.2011.11.018 (2012). 22340501

[b43] Wolburg H. & Lippoldt A. Tight junctions of the blood-brain barrier: development, composition and regulation. Vascul Pharmacol 38, 323–337 (2002). 12529927 10.1016/s1537-1891(02)00200-8

[b44] Gantress J., Maniero G. D., Cohen N. & Robert J. Development and characterization of a model system to study amphibian immune responses to iridoviruses. Virology 311, 254–262 (2003). 12842616 10.1016/s0042-6822(03)00151-x

[b45] Haislip N. A., Gray M. J., Hoverman J. T. & Miller D. L. Development and disease: how susceptibility to an emerging pathogen changes through anuran development. PloS one 6, e22307, doi: 10.1371/journal.pone.0022307 (2011). 21799820 PMC3142128

[b46] Grayfer L., De Jesus Andino F. & Robert J. Prominent amphibian (Xenopus laevis) tadpole type III interferon response to the frog virus 3 ranavirus. J Virol 89, 5072–5082, doi: 10.1128/jvi.00051-15 (2015). 25717104 PMC4403449

[b47] Singh M. V. . Characterization of platelet-monocyte complexes in HIV-1-infected individuals: possible role in HIV-associated neuroinflammation. J Immunol 192, 4674–4684, doi: 10.4049/jimmunol.1302318 (2014). 24729609 PMC4011982

[b48] Toborek M. . Mechanisms of the blood-brain barrier disruption in HIV-1 infection. Cell Molec Neurobiol 25, 181–199 (2005). 15962513 10.1007/s10571-004-1383-x PMC11529531

[b49] Persidsky Y., Ramirez S. H., Haorah J. & Kanmogne G. D. Blood-brain barrier: structural components and function under physiologic and pathologic conditions. J Neuroimmune Pharmacol 1, 223–236 (2006). 18040800 10.1007/s11481-006-9025-3

[b50] Diamond M. S. & Klein R. S. West Nile virus: crossing the blood-brain barrier. Nat Med. 10, 1294–1295, doi: 10.1038/nm1204-1294 (2004). 15580248

[b51] McGavern D. B. & Kang S. S. Illuminating viral infections in the nervous system. Nat Rev Immunol 11, 318–329, doi: 10.1038/nri2971 (2011). 21508982 PMC5001841

[b52] Dittrich S. . Blood-Brain Barrier Function and Biomarkers of Central Nervous System Injury in Rickettsial Versus Other Neurological Infections in Laos. Am J Trop Med Hyg 93, 232–237, doi: 10.4269/ajtmh.15-0119 (2015). 26055741 PMC4530739

[b53] Marr S. . Localization and differential expression of activation-induced cytidine deaminase in the amphibian Xenopus upon antigen stimulation and during early development. J Immunol 179, 6783–6789 (2007). 17982068 10.4049/jimmunol.179.10.6783

[b54] Robert J., Gantress J., Cohen N. & Maniero G. D. Xenopus as an experimental model for studying evolution of hsp–immune system interactions. Methods 32, 42–53 (2004). 14624877 10.1016/s1046-2023(03)00186-5

